# A surrogate in vitro experimental model for off-label drug repurposing: inhibitory effect of montelukast on bovine respiratory syncytial virus replication

**DOI:** 10.1186/s12985-025-02647-4

**Published:** 2025-02-15

**Authors:** Hanne Nur Kurucay, Zafer Yazici, Vahide Bayrakal, Bahadir Muftuoglu, Emre Ozan, Cuneyt Tamer, Seda Gozel, Gerald Barry, Mahir Igde, Semra Okur-Gumusova, Harun Albayrak, Ahmed Eisa Elhag, Huseyin Baskin

**Affiliations:** 1 Department of Virology, Faculty of Veterinary Medicine, Ondokuz Mayıs University, Samsun, Turkey; 2 Department of Quality Improvement in Healthcare and Accreditation, Institute of Health Sciences, Dokuz Eylül University, Izmir, Turkey; 3 Laboratory of Forensic Microbiology and Biological Defense, R & D and Service, Dokuz Eylül University Hospital, Izmir, Turkey; 4 Department of Experimental Animals, Faculty of Veterinary Medicine, Ondokuz Mayıs University, Samsun, Turkey; 5 Veterinary Science Center, School of Veterinary Medicine, University College of Dublin, Dublin, Ireland; 6 Department of Pediatric Allergy, Faculty of Medicine, Istinye University, Istanbul, Turkey; 7 Institute of Biochemistry and Biophysics, Polish Academic of Sciences, Warsaw, Poland; 8 Department of Preventive Medicine and Clinical Studies, Faculty of Veterinary Sciences, University of Gadarif, Al Qadarif, Sudan; 9 Department of Medical Microbiology, Faculty of Medicine, Dokuz Eylül University, Izmir, Turkey

**Keywords:** Antiviral, Epidemic, Leukotriene receptor antagonist, Montelukast sodium, Pandemic, RSV

## Abstract

**Background:**

Repurposing off-label drugs during epidemics or pandemics with unknown/known pathogens, particularly when their side effects and complications are already known, can be a strategic approach, as seen during the COVID-19 pandemic. Developing surrogate in vitro experimental models (passage-to-passage), which mimic epidemic/pandemic-like transmission (human-to-human), may enhance this repurposing process. This study evaluates montelukast sodium (MLS), a US FDA-approved leukotriene receptor antagonist for asthma, to explore its potential repurposing antiviral effects against bovine respiratory syncytial virus (BRSV), which has basic similarities to human respiratory syncytial virus (HRSV) as both belong to the *Pneumoviridae* family.

**Methods:**

An in vitro serial passage model was developed using MDBK cells infected with a local wild-type strain of BRSV (43TR2018). The cytotoxicity of MLS was assessed via the trypan blue exclusion method, identifying non-toxic concentrations. The impact of MLS on viral spread and infectivity was measured through TCID50 values over 10 passages. Viral loads were confirmed by nested RT-PCR and quantified using qPCR, while apoptosis, necrosis, and nitric oxide production were evaluated through staining and nitrite assays. Data were analyzed using ANOVA and Tukey's test (*p* < 0.05).

**Results:**

Control cells exhibited 97.16% viability, with 10 µM and 20 µM MLS concentrations maintaining viabilities of 89.2% and 87.3%, respectively. Viral titers significantly decreased at higher concentrations of MLS (up to 99.94% inhibition). Apoptosis rates decreased in MLS-treated cells, and live cell percentages improved, especially at 20 µM. Nitric oxide levels showed no significant differences across groups.

**Conclusion:**

MLS demonstrated a dose-dependent antiviral effect against BRSV, achieving 99% viral inhibition properties in MDBK cells. These promising results warrant further investigation into the antiviral mechanisms of MLS.

## Background

The number of current outbreaks of several viruses, including Ebola, zikavirus (ZIKV), West Nile, dengue (DENV), MERS-CoV, influenza A (IAV), SARS-CoV and SARS-CoV-2, increases, depending on variables such as increasing human population densities, intercontinental tourism, commercial acts, and global climate change [1]. Emergence of viruses with epidemic and pandemic potential threaten human populations due to the lack of available, appropriate and efficient treatment and protection. Although vaccines play a key role in the control of viral outbreaks, initially they may be insufficient. In a public health emergency such as swine flu and COVID-19 pandemics, developing broad-spectrum antivirals or virus-specific drugs, as well as novel strategies including searching off-label drugs, have vital importance alongside the vaccine. However, time to develop novel antivirals may be short [1, 2].

Developing a virus-specific drug and/or broad-spectrum antiviral is a long-running process that focuses on identifying viral proteins that drugs can target while limiting potential host cytotoxicity. Developing virus-specific therapeutics may be simpler and more straightforward than pursuing broad-spectrum antivirals [2].

Currently, strategies to develop antiviral drugs takes two approaches. The first directly targets the viruses, including inhibitors of viral attachment, entry, and uncoating, as well as protease, polymerase, reverse transcriptase, and other viral molecules. The second focuses on drugs that affect the host-cell machinery essential for viral infection and replication [2, 3]. Nowadays, researchers exhaustively discover and identify the biological molecules that have antiviral features, but the process is challenging and time-consuming. Repurposing off-label drugs may be one of the best alternative approaches to an effective therapy for viral diseases [4].

Cysteinyl leukotrienes (CysL-Ts) play an important role in the regulation of vascular leakage, and oedema, and also have effects on cytokine signaling in immune cells such as macrophages and lymphocytes. The lung alveolar macrophages are at the front line of defense in lower respiratory tract infections that can lead to lethal lung injuries. Type-1 alveolar epithelial cells produce leukotriene D4 (LTD_4_), which increases cellular uptake and virus replication by signaling cysteinyl receptors via CysL-Ts. In this context, the regulation of the alveolar epithelial cells by the alveolar macrophages through the suppression of LTD_4_ could be an important strategy to combat viral infection of the respiratory tract [4].

Montelukast sodium (MLS) is one of the CysL-T receptor antagonists inhibiting proinflammatory cytokines such as IL-1β, IL-6 and TNF-α [5]. It has always been thought to have an antiviral effect because of its high binding affinity to the viral protease required for RNA synthesis and replication [6]. Several studies assess its in vitro and in vivo effects against flaviviruses such as ZIKV and DENV [6], respiratory syncytial virus (RSV) [7] and IAV [8]. These studies report MLS inactivating these viruses early by damaging the lipid membrane and viral genome [6, 9]. The drug also inhibited expression of the viral genome in IAV [8].

Bovine respiratory syncytial virus (BRSV) is a member of the *Pneumoviridae* family genetically and antigenically similar to human respiratory syncytial virus (HRSV), as both are classified under the *Orthopneumovirus* genus, suggesting the potential use of BRSV in a repurposing surrogate study [10]. BRSV also causes respiratory disease in cattle, with clinical manifestations ranging from mild symptoms to fatal outcomes, influenced by multifactorial determinants [11]. To the best of our knowledge, this study is the first with MLS using BRSV as the model virus. Results demonstrated in vitro that MLS molecules lower the titer of BRSV.

## Materials and methods

### Cell lines, viruses and mutagen

Madin-Darby Bovine Kidney (MDBK) cell line and the local wild-type BRSV strain (GenBank no/Isolate: MT024767/43TR2018) were used in this study [11]. Both cell line and virus were provided from the cell line and virus stock collection of the Virology Department in the School of Veterinary Medicine of Ondokuz Mayis University. MDBK cells were grown in Dulbecco's Minimal Essential Medium (DMEM, Sigma, UK) supplemented with %10 fetal calf sera (FCS, Gibco, UK) and 1% antibiotic solution of penicillin and streptomycin (Sigma-Aldrich, USA). 43TR2018 was produced in freshly prepared MDBK cells by using DMEM supplemented with 1% FCS and stored at -80 C until use. MLS was kindly provided by Abdi-Ibrahim Pharmaceutical Company, Türkiye, dissolved in DMSO at 200 mM as a stock solution, and stored at − 20 C until use as 1 mL aliquots.

### Cell viability assay

An in vitro viability assay used the trypan blue exclusion staining method to determine the minimally toxic MLS concentration for MDBK cells. Briefly, 3.0 × 10^5^ per mL of MDBK cells were seeded in 24-well plates at 24 h and checked for 85% monolayer status on the day of the experiment. The different concentrations of MLS changing between 2.5, 5, 7.5, 10, 20, 40, 80 and 100 µM were prepared from stocked 1 ml of MLS in DMEM. The medium was removed from wells and replaced in duplicate with 1000 µl of each drug concentration. The plates were incubated at 37 °C for 24 h in a humidified chamber with 5% CO_2_. Each MLS concentration was then removed from the corresponding wells. Following adding 100 µl of 0.25% trypsin to each well to detach cells, plates were incubated for 1 min at 37 °C. The detached cells were collected from wells corresponding to each concentration. After gently pelleting using low-speed centrifugation, cells were resuspended in 20 µl of DMEM. A 10 µl aliquot of resuspended cells was mixed with an equal volume of 0.4% trypan blue, loaded in cell counting slides and then read using an automated cell counter (TC20, Bio-Rad, UK). The viability of cells for each MLS concentration was assessed as percentages based on the proportion of live cells to total cells.

### Drug treatment and 50% tissue culture infective dose (TCID_50_) assay

In this study, all passages and the corresponding viral titer assays were performed three times at different time intervals. To determine the antiviral effect of MLS, BRSV inoculations under pressure with selected non-cytotoxic concentrations of drug and without drug pressure were synchronously conducted using different 24-well plates including freshly prepared MDBK cells. Furthermore, a medium containing non-cytotoxic MLS concentrations was freshly prepared for each of the 10 passages performed in the study.

Briefly, three 24-well plates were seeded with 3 × 10^5^ MDBK cells per well, and incubated at 37 °C for 24 h. Optimal non-toxic drug concentrations were also freshly prepared on the virus inoculation day by diluting in DMEM. In two of the plates, the medium wells were aspirated. Cells were treated with 1 mL of selected non-cytotoxic MLS concentrations and left at 37 °C for 2 h for drug uptake. The media containing MLS were removed from all wells. Cells were infected with BRSV and incubated at 37 °C for 2 h, and then inocula were removed from cells after virus incubation. The virus was placed under pressure by adding a medium containing selected MLS concentrations to all wells at 37ºC in humidified 5% CO_2_ for 72 h with daily checking. As a positive control, the virus without the drug (∆BRSV) was also passaged in the growth medium in another plate via the same procedure and conditions above. The inocul**a** were collected from wells corresponding to 24, 48 and 72 h post-infection and stored at − 80 °C for the next passages and the experiments. The procedure was consecutively repeated 10 times.

At the end of each passage, the infectivity assay was performed in three 96-well plates using the 50% tissue culture infective dose (TCID_50_) method to compare the infectivities of BRSV with and without drug pressure. Inoculums of each passage obtained from 72 h were used for this assay. Briefly, we first added 100 µl of DMEM supplemented with 2% FCS to every well of three 96-well plates. 11 µl of BRSV with and without drug pressure, were added to the top wells in quadruplicate, followed by a ten-fold dilution descending to the end of the plate. After dilution, 50 µl of MDBK cells were added to each well, resulting in 4 × 10^4^ cells/well. The plates were incubated for 72 h at 37 °C before reading CPE and calculating the titer according to the method of Reed and Muench (1938) [12]. Titers were expressed as log_10_ TCID_50_/ml.

### Cloning

We subcloned the N gene of BRSV into a pGEMT easy vector as described by Tamer et al. [13] to make a standardization for the absolute quantification of BRSV-RNA copies in the infected MDBK cells. This process was performed by RT-PCR with the forward (5′-GCAATGCTGCAGGACTAGGTATAAT-3′) and the reverse primers (5′-ACACTGTAATTGATGACCCCATTCT-3′) using the iTaq Universal Probes OneStep Kit (BioRad, Cat No: 1725141) [14]. Following the preparation of a 25 µL mixture consisting of 12.5 µL 2 × iTaq buffer, 320 µM of each primer and 5 µL template RNA, the PCR reaction profile used the following PCR conditions: a cycle for 10 min at 50 °C, a cycle for 3 min at 95 °C, 40 cycles for 7 s at 95 °C, and 10 s at 59 °C. At the end of PCR conditions for amplification, 10 µL PCR product corresponding 124 bp fragment was run on 1% agarose gel containing 0.5 µg/mL ethidium bromide for 40 min and evaluated under UV. Amplicon was cloned into the pGEM-T easy vector system (Promega, Cat No: A1380) using the T4 DNA ligase via blunt-end ligation. JM109 E. coli cells were used for transformation.

Cloning and transformation were completed according to the manufacturer's manual. Briefly, a 10 μL ligation mixture was prepared as 5 μL 2 × rapid ligation buffer (400 mM Tris–HCl, 100 mM MgCl_2_), 2 μL T4 DNA ligase (3 Weiss U/μL), 100 ng purified BRSV N gene amplicon, 50 ng pGEM-T Easy plasmid, and incubated overnight at 4 °C. For transformation, 50 μL of JM109 competent *E. coli* (1 × 108 CFU/µg DNA) was put into a 10 μL volume of ligation mixture, gently pipetted and incubated for 30 min on ice. The cells were shocked at 42 °C for 50 s, then placed again on ice and incubated for 2 min. 150 μL of SOC medium (2% tryptone, 0.5% yeast extract, 10 mM NaCl, 2.5 mM KCl, 10 mM MgCl_2_, 10 mM MgSO_4_ and 20 mM glucose) at room temperature was added to the mixture and incubated at 37 °C at 150 rpm in a shaking incubator for 90 min. The broth was spread on an LB agar (10 g Bacto tryptone, 5 g Bacto-yeast, 10 g NaCl, 15 g agar and 100 μg/mL ampicillin) plate and incubated at 37 °C overnight. White colonies containing N gene insert were picked and cultivated in 2 × YT liquid broth (16 g/L tryptone, 10 g/L yeast extract, 5 g/L NaCl and 100 μg/mL ampicillin) for 16 h. Plasmids were extracted from 2 × YT liquid broth using a GeneJET plasmid miniprep kit (Thermo, Cat No: K0503) and then stored at -20 °C.

### Calculation of viral loads

We measured the level of BRSV G gene inserted into the pGEM-T easy vector, using a spectrophotometer. The number of viral copies per µL was calculated using the formula below. The plasmid was reconstituted ten times in log10 dilutions used as the standard in qPCR to calculate the viral load in MDBK cells.

$${\text{Viral}}\,{\text{copy}}\,\left( {{1 }\mu {\text{L}}} \right) = \frac{{{\text{Amount}}\,\left( {{\text{nguL}}} \right) \times {6}.0{22} \times {1}0^{{{23}}} }}{{\left[ {{\text{Plasmid}}\, + \,{\text{insert }}\,\left( {{\text{bp}}} \right)} \right] \times {1} \times {1}0^{{9}} \times {65}0}}$$

### Nested RT-PCR assays

The viral RNA was extracted from infected cell culture lysates of the selected passages of BRSV with and without MLS pressure using a commercial GeneJET RNA purification kit (Thermo-Fischer, UK) according to the manufacturer’s instructions. The extracted RNAs were eluted in 75 µL elution buffer and stored at -80ºC until used.

The nested- RT-PCR for the G gene of BRSV, described by Vilcek et al. [15], was performed to confirm selected passages of BRSV with and without MLS pressure using primer sets given in Table 1 with amplicon sizes. The first round of RT-nested PCR was conducted in 50 µL of a reaction mixture that contained 10 µL of 5 x reaction buffer, 1 µL of dNTP, 2 µL of primers (B5A and B6A),6A), 1 µL of an enzyme, 5 µL of a template and 29 µL of RNAse free water. Amplification was performed with Multigene Thermal Cycler (Cleaver Scientific Ltd, UK) of 30 min at 50 °C for reverse transcription and 15 min at 95 °C, followed by 35 cycles at 95 °C for 45 s, 50 °C for 45 s, 72 °C for 60 s, and finally a cycle at 72 °C for 10 min. The second round of PCR was also carried out in a final volume of 50 µL containing 5 µL of the first round RT-PCR product, 10 µL of 5 x buffer, 2 µL of each primer (B7A and B8), 1 µL of dNTP, 1 µL of RT enzyme, and 29 µL of RNAse-free water. The cycling conditions employed for the second round of PCR were as follows: 1 min at 95 °C and 45 s at 95 °C, then 35 cycles at 94 °C for 45 s, 50 °C for 45 s, 72 °C for 1 min, and finally a cycle at 72 °C for 10 min. Ten microliters out of each PCR product was loaded onto a 1.5% agarose gel stained with ethidium bromide running at 100 V for 40 min and visualized by the Quantum gel imaging and documentation system (Vilber Lourmat, Collegien, France).

**Table 1:** The qPCR and nested RT-PCR primers/probe sequences of BRSV that were used in this study

Primers or probe	Sequence 5′–3′	The product size(bp)	References
F primer*	GCAATGCTGCAGGACTAGGTATAAT	124	[14]
R primer*	ACACTGTAATTGATGACCCCATTCT		
Probe*	FAM-ACCAAGACTTGTATGATGCTGCCAAAGCA-TAMRA		
B5A**	CCA CCC TAG CAA TGA TAA CCT TGA C	603	[15]
B6A**	AAG AGA GGA TGC (T/C)TT GCT GTG G		
B7A**	CAT CAA TCC AAA GCA CCA CAC TGT	371	
B8**	GCT AGT TCT GTG GTG GAT TGT TGT C		

*Real-time RT-PCR primers and probe for the N gene of BRSV

**Nested -PCR primers for the G gene of BRSV

### Morphologic assessment of apoptosis and necrosis using Hoechst 33342/propidium iodide nuclear staining and fluorescence microscopy

All cells were stained with Hoechst 33342 and propidium iodide (PI) and were visualized using fluorescence microscopy (Leica DMIL, Leica Microsystems Type). Cells were incubated for 15 min at 37 °C with Hoechst 33342 dye (5 mg/mL in PBS) and then washed three times in phosphate-buffered saline (PBS). Propidium iodide (50 mg/mL from a 1 mg/mL stock in PBS) was added just before microscopy. A minimum of 100 cells in three areas were counted and classified as follows: (1) live cells (normal nuclei: blue chromatin with organized structure); (2) membrane intact apoptotic cells (bright blue chromatin that is highly condensed, marginated, or fragmented); (3) membrane-permeable apoptotic cells (bright red chromatin, highly condensed, or fragmented); (4) necrotic cells (red, enlarged nuclei with smooth normal structure); and (5) pyknotic/ necrotic cells (bright red, slightly condensed nuclei sometimes divided into 2–3 spheres) [16].

### Nitrite determination

Rapid oxidation of nitric oxide (NO) generates nitrite. It is stable and its accumulation in the culture medium reflects the amount of NO produced. Aliquots of 100 µl cell culture supernatants were mixed with equal volumes of Griess reagent (equal volumes of 0.1% *N*-(1-naphthyl)-ethylenediamine dihydrochloride and *p*-aminobenzene sulfanilamide diluted in 1.5% phosphoric acidin in a 96-well microtitre plate) (Maxisorb Immunoplate, Nunc). After 10 min of incubation at room temperature, the absorbance at a wavelength of 540 nm was measured in a microplate reader (Thermo, spectrophotometric model system). A range of twofold dilutions of sodium nitrite (0.05–100 μM) in DMEM medium was run in each assay to generate a standard curve. All experiments were repeated at least 3 times [17].

### Statistical analysis

One-way analysis of variance (one-way ANOVA) evaluated the differences among the means of viral titers and copies for all consecutive passages, along with the percentages of cytotoxicity values, apoptosis, necrosis and nitric oxide responses for cells. Tukey's multiple comparison test evaluated the percentages of apoptosis, necrosis and nitric oxide responses of experiment groups, including control groups. GraphPad Prism software version 5.0 (GraphPad 5.0 software, USA) was used for all statistical analysis, and a *p < 0.05* value was considered significant.

## Results

### Cytotoxicity assays

**Fig. 1:**
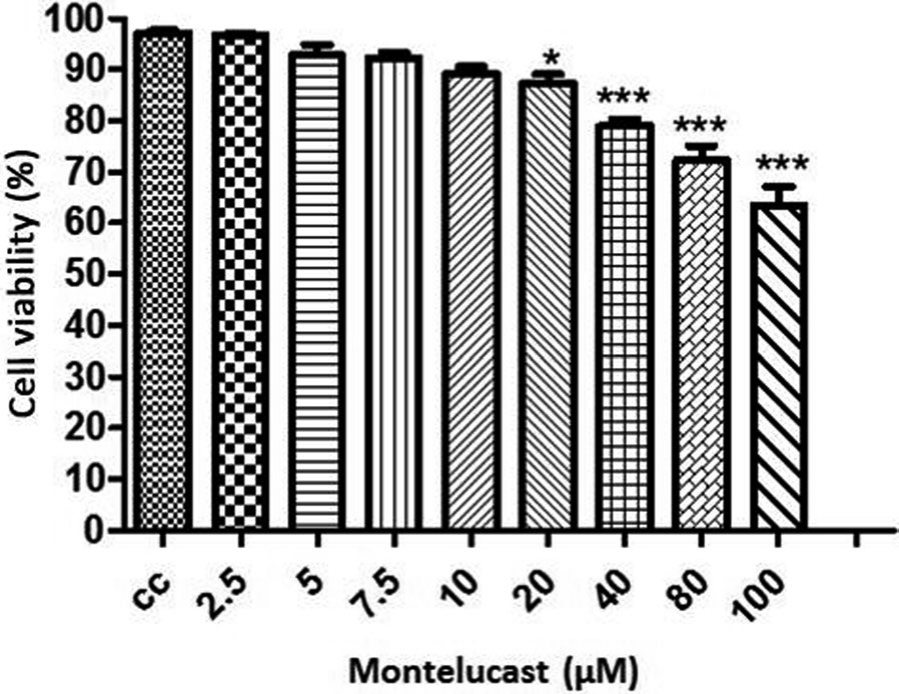
Chart showing the cell viability percentage of MDBK under the pressure of different concentrations of MLS. (*): The highest statistically significant concentration of MLS (20 µM), (***): 40 µM, 80 µM and 100 µM concentrations of MLS were found statistically high cytotoxic effect on MDBK cells, whereas the cell viability percentage was calculated with more than 80% for 10 µM and 20 µM of MLS, as 89.2% ± 1.33 and 87.3% ± 1.7, respectively and evaluated statistically significant (*p* < 0.05)

The percentage of viability for cell control was 97.16% ± 0.44, whereas it was calculated with more than 80% for 10 µM and 20 µM, 89.2% ± 1.33 and 87.3% ± 1.7, respectively, and statistically significant (*p* < *0.05*). The cell viability also continued to diminish between 40 µM and 100 µM concentrations and calculated as 78.96% + 1.33 and 63.46% + 3.58, respectively. The results were statistically highly significant (Fig. 1). The optimal concentrations for MLS used in this study were 10 µM and 20 µM.

### Montelukast suppresses the spreading of BRSV in MDBK cells with limited effects on viral titer

To investigate the antiviral effects of MLS on BRSV infection in vitro, we suppressed the virus by using 10 µM and 20 µM concentrations of the drug. Virus under drug pressure were marked as _MLS_▲_10_BRSV and _MLS_▲_20_BRSV, whereas virus without drug pressure was marked as ∆BRSV. Consecutive 10 passages were synchronously conducted by ∆BRSV, _MLS_▲_10_BRSV and _MLS_▲_20_BRSV and monitored for 72 h for each passage. The beginning of CPE in each passage was observed during the first 24 h after virus inoculations.

**Fig. 2:**
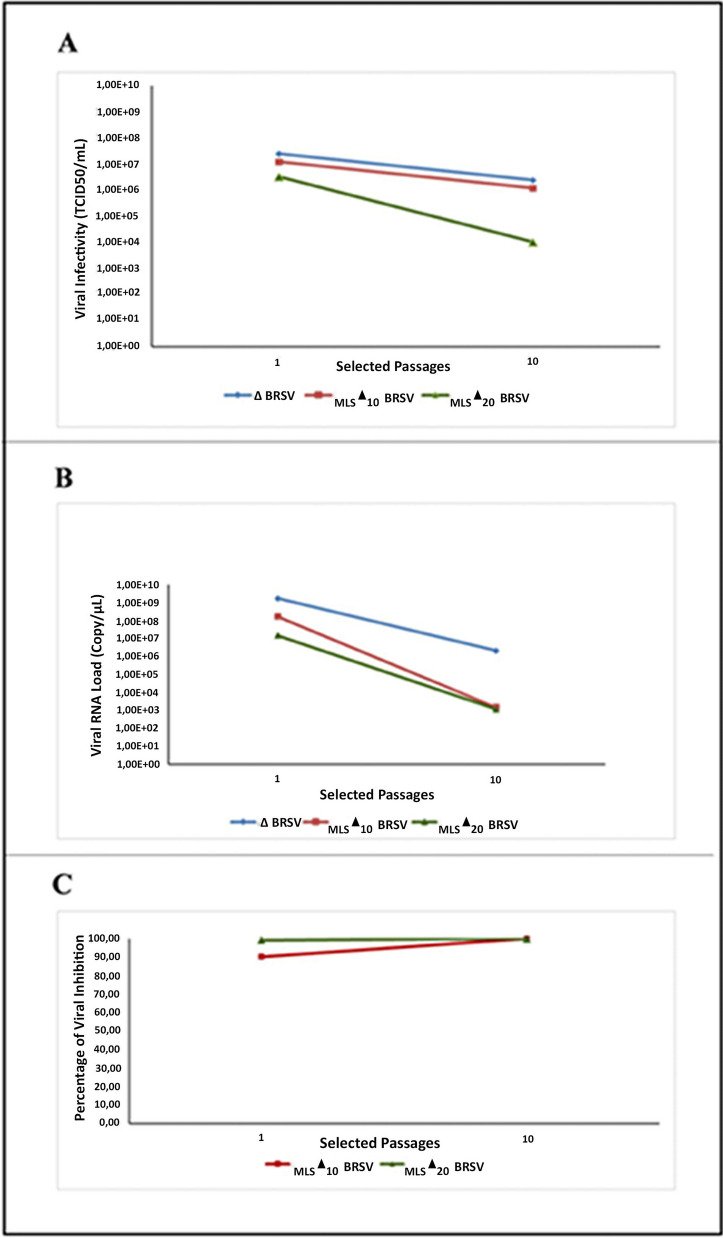
Viral titer—Viral copy—Viral inhibition values following 10 and 20 μM MLS application in the first and tenth passages of 72 h of incubation: **A** Viral titer according to the Median Tissue Culture Infectious Dose (TCID_50_),** B** Viral copy/μL,** C** Percent of viral inhibition

In the first passage, the pervasive CPE was observed in the MDBK monolayers 72 h after virus inoculation. At the end of 10 passages, the spread of BRSV was reduced by both _MLS_▲_10_BRSV and _MLS_▲_20_BRSV. The inhibition of the viral spread was slightly more in _MLS_▲_10_BRSV and _MLS_▲_20_BRSV. The decrease in viral titers in each passage was assessed to determine viral infectivity. Viral titers under the pressure of 10 µM and 20 µM concentrations of MLS decreased compared with control virus (∆BRSV). Furthermore, we observed that the decrease in viral titers was in concordance with the reduction of CPE. A decrease in these values was determined in the 10th passage and the viral titers were found for _MLS_▲_20_BRSV, _MLS_▲_10_BRSV and ∆BRSV being 1.0 × 10^4^, 5.99 × 10^5^ and 2.99 × 10^6^, respectively (Fig. 2A). The at least 2 log reductions in the viral titers of _MLS_▲_10_BRSV and _MLS_▲_20_BRSV indicate inhibition of viral spread.

### PCR results and the determination of viral loads for selected passages

**Fig. 3:**
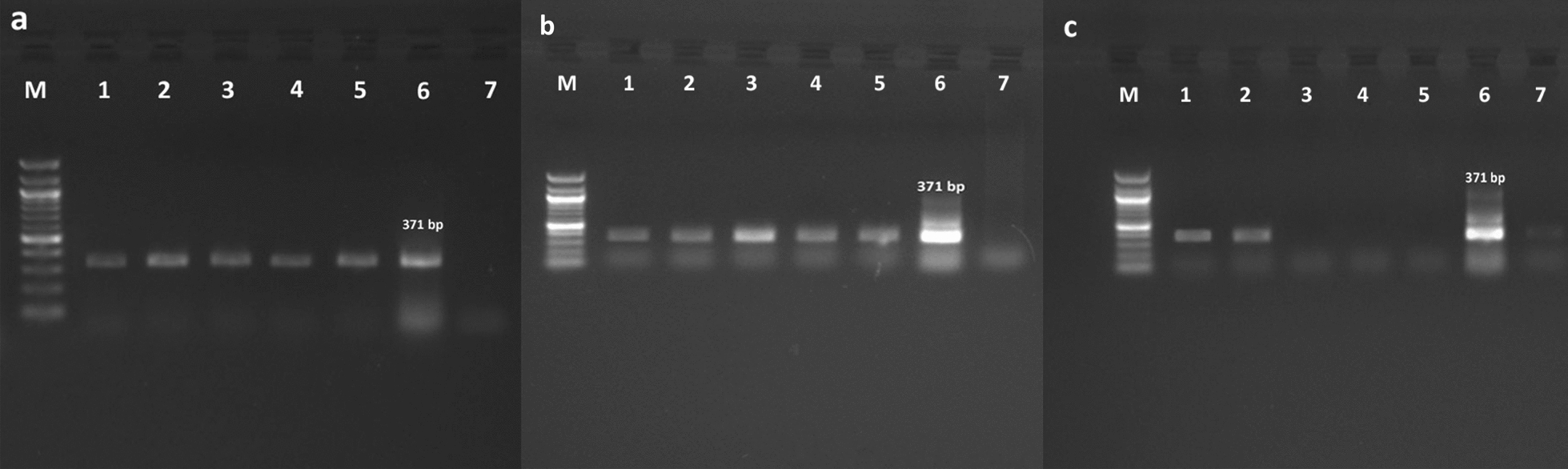
The nested RT-PCR results of the selected 1, 3, 5, 8 and 10th passages of ∆BRSV, _MLS_▲_10_BRSV and _MLS_▲_20_BRSV that were indicated between lanes 1 and 5, lane 6: positive control, lane 7: negative control and M: 100 bp ladder. **a** ∆BRSV refers to the virus without MLS, **b**_MLS_▲_10_BRSV refers to the virus under drug pressure with 10 µM /mL of MLS, and **c**_MLS_▲_20_BRSV refers to the virus under drug pressure with 20 mM/mL of MLS

To the nested RT-PCR, 1, 3, 5, 8 and 10 of the passages of ∆BRSV, _MLS_▲_10_BRSV and _MLS_▲_20_BRSV were selected. BRSV RNA was found in all selected passages. As depicted in Fig. 3, the expected amplicon size of 371 bp corresponding to the G gene region of the virus was also visualized by gel electrophoresis. Both the strong and clear bands were observed in the 1, 3 and 5th passages of ∆BRSV _MLS_▲_10_BRSV and _MLS_▲_20_BRSV. The band corresponding to 371 bp for the 10th passage was much fainter for _MLS_▲_20_BRSV compared to that for _MLS_▲_10_BRSV as well as ∆BRSV.

Viral loads were calculated for _MLS_▲_10_BRSV, _MLS_▲_20_BRSV and ∆BRSV whether or not MLS inhibited the virus, as depicted in Fig. 2B. In the first passage, the viral loads per µl were calculated for ∆BRSV, _MLS_▲_10_BRSV and _MLS_▲_20_BRSV as 1.73 × 10^9^, 1.71 × 10^8^ and 1.06 × 10^7^ respectively. We found these values in the 10th passages for ∆BRSV, _MLS_▲_10_BRSV and _MLS_▲_20_BRSV as 2.16 × 10^6^, 1.54 × 10^3^ and 1.18 × 10^3^ respectively. These results indicated that 10 µM and 20 µM concentrations of MLS reduced BRSV viral copy number by 1402 (99.92%) and 1830 (99.94%) fold as well as approximately 99.90% viral inhibition, respectively (Fig. 2C). When _MLS_▲_10_BRSV and _MLS_▲_20_BRSV were compared with ∆BRSV as of the first passage, a gradual reduction of viral loads, indicating an antiviral effect, was observed.

### Apoptosis, necrosis, live cells percentages and nitric oxide responses of MLS

#### First passage

##### Controls

**Fig. 4:**
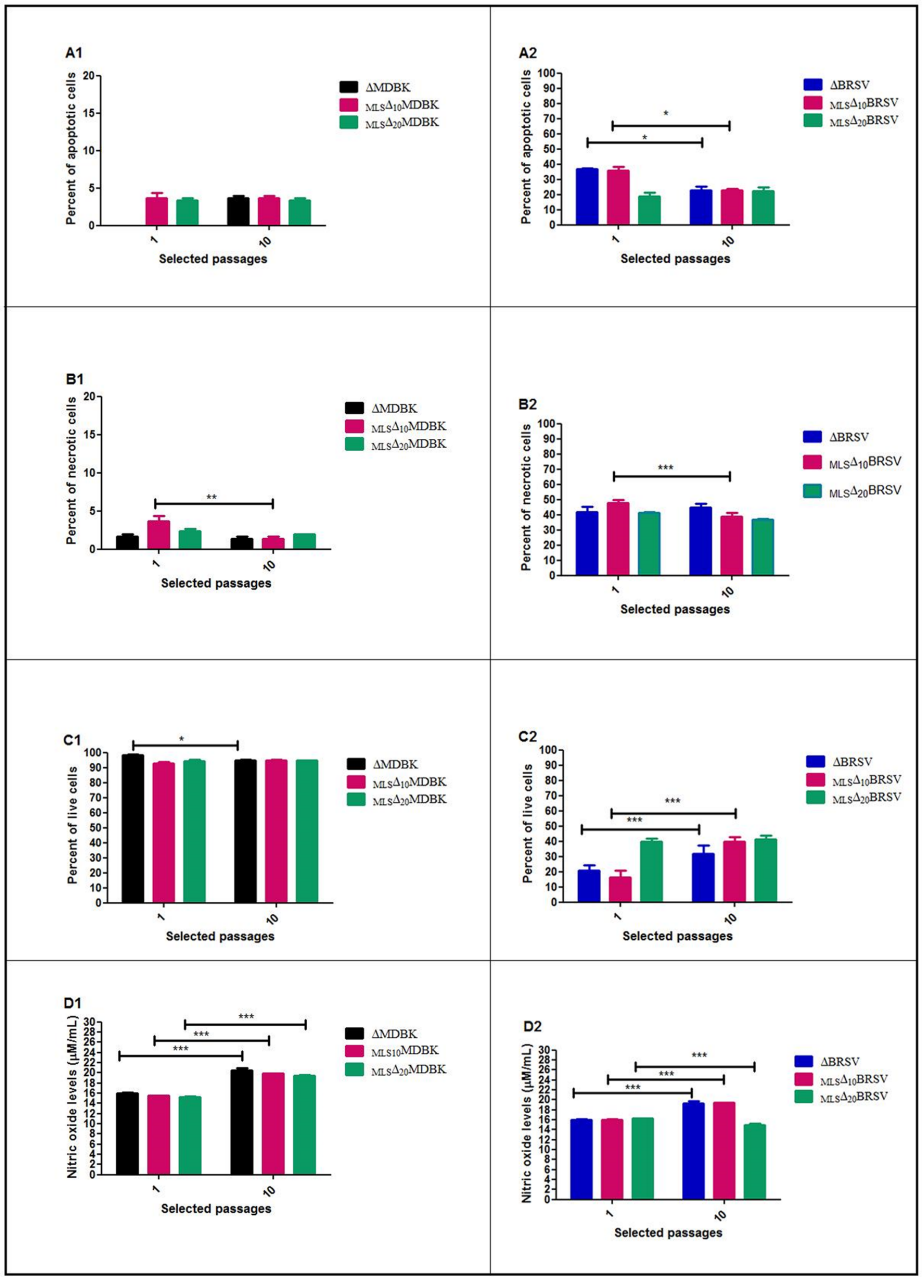
Percents of apoptosis, necrosis, live cells and nitric oxide responses of selected passages: **A1**, **B1**, **C1** and **D1** graphics show the inter-experimental control groups. Control groups are designed to question the possible cytotoxic effect of the MLS-used doses (_MLS_▲_10_MDBK, _MLS_▲_20_MDBK) in the first and tenth passages compared to cells without drug pressure (ΔMDBK). Apoptosis/necrosis/live cells percents and nitric oxide responses of these groups show us that “there is no possible stress of cells under drug pressure which might affect the results of experiments”. **A2**, **B2**, **C2**, **D2** graphics show the experimental groups, which are designed to question the possible antiviral and immunoregulatory effect of MLS on BRSV depending on two doses, 10 μM and 20 μM (_MLS_▲_10_BRSV, _MLS_▲_20_BRSV), (Since all experimental results take up a lot of space, only graphs of the initial and final passage values are presented). *p < 0.05, **p < 0.001, ***p < 0.0001

1. In the first passage, the apoptosis response of cells without drug pressure marked as ΔMDBK was 0.00 ± 0.00% (p < 0.05). Apoptosis response of cells with 10 µM drug pressure (_MLS_▲_10_MDBK) was 3.66 ± 1.15%, whereas 3.33 ± 0.57% (NS) was recorded in cells with 20 µM drug pressure (_MLS_▲_20_MDBK) (Fig. 4A1).

*Control values showed that at the initial point of experiments, MLS had no stimulatory effect on apoptosis.

2. In the first passage, the necrosis response of ΔMDBK was 1.66 ± 0.57% (NS). Necrosis response of _MLS_▲_10_MDBK was 3.66 ± 1.15%, whereas it was 2.33 ± 0.57% (NS) in _MLS_▲_20_MDBK (Fig. 4B1).

*Control values showed that at the initial point of experiments, MLS had a negligible stimulatory effect on necrosis.

3. In the first passage, the live cell percentage of ΔMDBK was 98.44 ± 0.43% (NS) (*p* < 0.05). Live cell percentage of _MLS_▲_10_MDBK was 92.66 ± 2.30% (NS), whereas it was 94.33 ± 1.15% (NS) in _MLS_▲_20_MDBK (Fig. 4C1).

*Control values showed that at the initial point of experiments, MLS had a negligible inhibitory effect on live cell percentages depending on MLS concentrations.

4. In the first passage, the nitric oxide response of ΔMDBK was 15.96 ± 0.28 µM/mL (*p* < 0.0001). Nitric oxide response of _MLS_▲_10_MDBK was 15.53 ± 0.11 µM/mL (*p* < 0.0001), whereas it was 15.26 ± 0.11 µM/mL (p < 0.0001) in _MLS_▲_20_MDBK (NS) (Fig. 4D1).

*Control values showed that at the initial point of experiments, MLS had a negligible stimulatory effect on nitric oxide response.

##### Experiment groups

1. In the first passage, the apoptosis response of cells infected with ΔBRSV was 37.00 ± 1.00% (*p* < 0.05). Apoptosis response decreased to 18.66 ± 4.50% in cells infected with _MLS_▲_20_ BRSV (p < 0.0001), whereas it was 36.00 ± 4.00% in cells infected with _MLS_▲_10_ BRSV (*p* < 0.05) (Fig. 4A2).

*MLS had an inhibitory effect on “BRSV-induced apoptosis” depending on the concentration of MLS.

2. In the first passage, the necrosis response of ΔBRSV infected cells was 42.00 ± 6.00% (*p* < 0.0001). Necrosis response decreased to 41.33 ± 1.15% in _MLS_▲_20_BRSV infected cells (*p* < 0.0001), but increased to 47.66 ± 4.04% in _MLS_▲_10_BRSV infected cells (*p* < 0.0001)(Fig. 4-B2).

*MLS had an inhibitory effect on “BRSV-induced necrosis” depending on the concentration of MLS.

3. In the first passage, the live cell percentage of ΔBRSV infected cells was 21.00 ± 5.56% (*p* < 0.05). Live cell percentage decreased to 16.33 ± 7.76% in _MLS_▲_10_ BRSV infected cells (*p* < 0.0001), whereas it increased to 40.00 ± 3.60% (*p* < 0.0001) in _MLS_▲_20_BRSV infected cells (Fig. 4C2).

*At the initial point of experiments, MLS had a “clear-cut protective” effect on live cell percentages depending on the concentration of MLS.

4. In the first passage, the nitric oxide response of ΔBRSV infected cells was 16.03 ± 0,28 µM/mL (*p* < 0.0001). Nitric oxide response of _MLS_▲_10_MDBK infected cells was 16.03 ± 0,20 µM/mL (*p* < 0.0001), whereas it was 16,23 ± 0,11 µM/mL (*p* < 0.0001) in _MLS_▲_20_MDBK infected cells (NS) (Fig. 4D2).

*At the initial point of experiments, MLS had no effect on nitric oxide response in BRSV-infected cells, whereas BRSV also had no effect on nitric oxide response.

#### Tenth passage

##### Controls

1. At the tenth passage, the apoptosis response of ΔMDBK cells was 3.66 ± 0.57% (NS). Apoptosis response of _MLS_▲_10_MDBK was 3.66 ± 0,57%, whereas it was 3.33 ± 0.57% (NS) in _MLS_▲_20_MDBK. (Fig. 4A1).

*Control values showed that MLS had no stimulatory effect of apoptosis at tenth passage.

2. At the tenth passage, the necrosis response of ΔMDBK cells was 1.33 ± 0.57% (NS). Necrosis response of _MLS_▲_10_MDBK was 1.33 ± 0.57%, whereas it was 2.00 ± 0.00% (NS) in _MLS_▲_20_MDBK (Fig. 4B1).

*Control values showed that MLS had a negligible stimulatory effect on necrosis at tenth passage.

3. At the tenth passage, the live cell percentage of ΔMDBK cells was 95.00 ± 0.43% (*p* < 0.05). Live cell percentage of _MLS_▲_10_MDBK was 95.00 ± 1,00% (NS), whereas it was 95.00 ± 0.00% (NS) in _MLS_▲_20_MDBK (Fig. 4C1).

*Control values showed that at the tenth passage of experiments, MLS had no effect on live cell percentages.

4. At the tenth passage, the nitric oxide response of ΔMDBK cells was 20.53 ± 0.63 µM/mL (*p* < 0.0001). Nitric oxide response of _MLS_▲_10_MDBK was 19.83 ± 0.05 µM/mL (*p* < 0.0001), whereas it was 19.46 ± 0.30 µM/mL (*p* < 0.0001) in _MLS_▲_20_MDBK (NS) (Fig. 4D.)

*Control values showed that MLS had a negligible effect on nitric oxide response.

##### Experiment groups

1. At the tenth passage, the apoptosis response of ΔBRSV cells was 23.00 ± 4.35% (*p* < 0.05), whereas it was 22.66 ± 2.3% (*p* < 0.0001) in _MLS_▲_10_BRSV and 22.33 ± 4.72% (*p* < 0.0001) in _MLS_▲_20_BRSV cells (Fig. 4A2).

*At the tenth passage, MLS had no effect on apoptosis response.

2. At the tenth passage, the necrosis response of ΔBRSV cells was 44.66 ± 4.50% (*p* < 0.0001). Necrosis response decreased to 38.66 ± 4.72% (*p* < 0.0001) in _MLS_▲_10_BRSV cells and 37.00 ± 1.00% in _MLS_▲_20_BRSV cells (*p* < 0.0001) (Fig. 4B2).

*At the tenth passage, MLS had a “clear cut inhibitory effect” on necrosis response.

3. At the tenth passage, the live cell percent of ΔBRSV cells was 32.00 ± 8.88% (*p* < 0.001). Live cell percent increased to 40.00 ± 4.58% in _MLS_▲_10_ BRSV cells (*p* < 0.0001) whereas it increased to 41.33 ± 4.16% (*p* < 0.0001) in _MLS_▲_20_BRSV cells (Fig. 4C2).

*At the tenth passage of experiments, MLS had a “cell protective effect” on BRSV infected MDBK cells depending on MLS concentrations.

4. At the tenth passage, the nitric oxide response of ΔBRSV cells was 19.33 ± 0.63 µM/mL (*p* < 0.0001). Nitric oxide response of _MLS_▲_10_MDBK was 19.76 ± 0.11 µM/mL (*p* < 0.0001) whereas it was 14.96 ± 0.40 µM/mL (*p* < 0.0001) in _MLS_▲_20_MDBK (NS) (Fig. 4D2).

*At the tenth passage of experiments, MLS had an inhibitory effect on nitric oxide response in BRSV infected cells depending on MLS concentrations whereas BRSV alone had no effect on nitric oxide response.

**Fig. 5:**
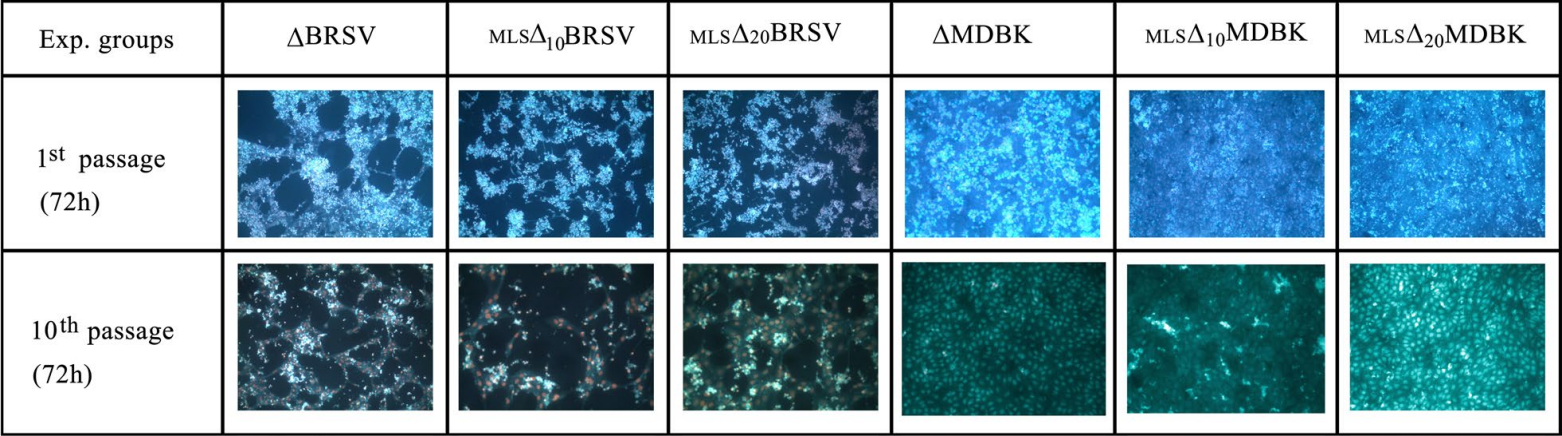
Apoptosis/necrosis presentations of first and tenth passages of 72-h incubation: Cells were stained with Hoechst 33342 (apoptosis) and propidium iodide (PI) (necrosis) and were visualized by fluorescence microscopy (Leica DMIL, Leica Microsystems Type)

ΔBRSV alone had a clear cytopathic effect (CPE) on MDBK cells, at the first and also at the tenth passages. This cytopathic effect decreased when two different doses of MLS were introduced (_MLS_▲_10_BRSV, _MLS_▲_20_BRSV), which had no cytotoxic effect on MDBK cells (Fig. 5).

## Discussion

Repurposing off-label drugs may be a smart strategy when side effects and complications are already known in an epidemic/pandemic situation [18]. Benchside solutions should be planned carefully. Use of an in vitro experimental surrogate model of epidemic/pandemic infection fits the aim of translational medicine, from-benchside-to-bedside and from-bedside-to-benchside [19].

As RSV infection progresses, necrosis and disorganized proliferation of bronchiolar epithelium and destruction of epithelial cells become hallmarks of bronchiolitis. Another important pathological characteristic of RSV infection is submucosal edema and mucus hypersecretion, which combine with cell debris and inflammatory cells, causing bronchiolar obstruction, air trapping, and emphysema.

Impaired gas exchange results in hypoxemia and, in more severe cases, the need for supportive respiratory therapy. In severe RSV, inflammatory cells accumulate in the airway mucosa as inflammatory regulators are continuously released and by viral replication affects the epithelial cells [20, 21].

MDBK is a continuous, stable epithelial cell line that originated from bovine kidney. RSV infection in epithelial cells triggers multiple signaling pathways, including NF-κB (nuclear factor kappa B) and nitric oxide. RSV is a strong activator of NF-κB, which is necessary for RSV infection-inducible inflammatory and immunoregulatory gene transcription. In a mouse model of infection, RSV activated NF-κB in the early phase of infection [22]. In another study, the inhibiton of NF-κB reduced cytokine production and clinical disease, although viral replication did not decrease [23]. Together, these findings suggest that activation of the host inflammatory response via NF-κB has a pivotal role in the immunopathogenesis of RSV infection [20]. On the contrary, we found that BRSV did not induce apoptosis, unlike in the previous studies.

MLS is a known US FDA approved leukotriene receptor antagonist drug used in the treatment and prevention of asthma [24], but clinical dosage of the drug is important because of possible side effects, especially in children [25]. Nitric oxide is an intercellular signaling molecule that regulates the normal (constitutive) and host defensive functions of the cells.

In this study, an in vitro serial passage method, which resembles in vitro epidemic/pandemic-like conditions, was used to assess a possible antiviral and host supportive regulatory effect of Montelukast against a wild-type strain of BRSV. We might say that Montelukast had a host preventive and antiviral effect on BRSV.

In vitro studies in the literature support the antiviral effect of MLS against viruses [6, 26, 27, 28]. Chen et al. reported that this antiviral effect changed between 2Log_10_ and 1.2log_10_, and was almost lost under the pressure of 10 µM MLS in particular [27].

The experiment results of this study were compatible with previous studies. Our team reported that both 10 µM (_MLS_▲_10_BRSV) and 20 µM (_MLS_▲_20_BRSV) doses of MLS had clear antiviral effects on BRSV in vitro with reducing viral titer (TCID_50_) at least 2log_10_ when compared to ΔBRSV. Furthermore, viral loads between ΔBRSV and _MLS_▲_10_BRSV and _MLS_▲_20_BRSV groups markedly differed. The comparisons between the 1st passage and the 10th passage indicated that viral loads were greatly decreased by the selected doses of MLS with over 99% viral inhibition. Sharp decreases in both viral load and viral titer in our study could be interpreted as depending on two consequences: a demolishing of the integrity of the virus due to the increasing release of BRSV-RNA inducted by MLS [27], and/or MLS targeting the lipid membrane of BRSV [6].

MLS can decrease extra-pulmonary manifestations in COVID-19, either directly through blocking Cys-LTRs in different organs or indirectly through inhibition of the NF-κB signaling pathway [29]. In addition, RSV is a potent activator of NF-κB [17] and MLS is a potent inhibitor of NF-κB. [30]. This study showed that MLS had a clear cut inhibitory effect on nitric oxide response, which is a marker of NF-κB activation [20].

After ten passages of 72 h post-infection, the percentage of viable cells in MDBK cells was not affected to a degree that would affect the experimental results. When we compared the control groups in the first and tenth passages, MLS had no cytotoxic effect on living cells at either dose (Fig. 4C1). Comparison of the control groups in the first and tenth passages indicated that nitric oxide responses remained at the levels of MDBK cells in both doses of MLS (Fig. 4D1). When the percentages of living cells in the first and tenth passages are compared, MLS may have had a protective effect on the cells, depending on dose (Fig. 4C1/C2). It was interesting to see that _MLS_▲_20_BRSV had a clear viral inhibition in the first and tenth passages, and live cell percentages were clearly higher at the initial point of viral infection (Fig. 4C2). Comparing the first passage with the tenth demonstrated that nitric oxide responses increased under BRSV pressure, although the viral copy number decreased. However, MLS also had a dose-dependent suppressive effect on nitric oxide responses, even in the presence of BRSV (Fig. 4D2).

## Conclusion

Results from this study indicated that MLS is a viral inhibitor, non-apoptotic, and mildly necrotic. MLS had a 99% antiviral effect on BRSV in MDBK cells and a protective role in MDBK cells. Consequently, antiviral effect pathways of MLS warrant further investigation.

## Data Availability

No datasets were generated or analysed during the current study.

## References

[1] Beyer RM, Manica A, Mora C. Shifts in global bat diversity suggest a possible role of climate change in the emergence of SARS-CoV-1 and SARS-CoV-2. Sci Total Environ. 2021;767:145413. 10.1016/j.scitotenv.2021.145413. 33558040 10.1016/j.scitotenv.2021.145413 PMC7837611

[2] Geraghty RJ, Aliota MT, Bonnac LF. Broad-spectrum antiviral strategies and nucleoside analogues. Viruses. 2021;13(4):667. 10.3390/v13040667. 33924302 10.3390/v13040667 PMC8069527

[3] Kausar S, Said Khan F, Ishaq-Mujeeb-Ur-Rehman M, Akram M, Riaz M, Rasool G, Hami, et al. A review: mechanism of action of antiviral drugs. Int J Immunopathol Pharmacol. 2021. 10.1177/20587384211002621. 33726557 10.1177/20587384211002621 PMC7975490

[4] Dey M, Singh RK. Possible therapeutic potential of cysteinyl leukotriene receptor antagonist montelukast in treatment of SARS-CoV-2-Induced COVID-19. Pharmacology. 2021;106(9–10):469–76. 10.1159/000518359. 34350893 10.1159/000518359

[5] Chakkarwar PR. Montelukast - its immuno-modulatory and antiviral action in COVID-19. J Evid Based Med Healthc. 2021;8(21):1731–2. 10.18410/jebmh/2021/327.

[6] Chen Y, Li Y, Wang X, Zou P. Montelukast, an anti-asthmatic drug, inhibits zika virus infection by disrupting viral integrity. Front Microbiol. 2020;10:3079. 10.3389/fmicb.2019.03079. 32082265 10.3389/fmicb.2019.03079 PMC7002393

[7] Fitzgerald D, Mellis C. Leukotriene receptor antagonists in virus-induced wheezing: evidence to date. Treat Respir Med. 2006;5(6):407–17. 10.2165/00151829-200605060-00006 17154670 10.2165/00151829-200605060-00006

[8] Landeras-Bueno S, Fernández Y, Falcón A, Oliveros JC, Ortín J. Chemical genomics ıdentifies the PERK-mediated unfolded protein stress response as a cellular target for ınfluenza virus ınhibition. MBio. 2016;7(2):e00085-e116. 10.1128/mBio.00085-16. 27094326 10.1128/mBio.00085-16 PMC4850254

[9] Gan HJ, Harikishore A, Lee J, Jeon S, Rajan S, Chen MW, Neo JL, Kim S, Yoon HS. Antiviral activity against Middle East Respiratory Syndrome coronavirus by Montelukast, an anti-asthma drug. Antiviral Res. 2021;185:104996. 10.1016/j.antiviral.2020.104996. 33309540 10.1016/j.antiviral.2020.104996 PMC7726485

[10] Valarcher JF, Schelcher F, Bourhy H. Evolution of bovine respiratory syncytial virus. J Virol. 2000;74(22):10714–28. 10.1128/jvi.74.22.10714-10728.2000. 11044116 10.1128/jvi.74.22.10714-10728.2000 PMC110946

[11] Yazici Z, Ozan E, Tamer C, Muftuoglu B, Barry G, Kurucay HN, Elhag AE, Cagirgan AA, Gumusova S, Albayrak H. Circulation of indigenous bovine respiratory syncytial virus strains in turkish cattle: the first isolation and molecular characterization. Animals. 2020;10(9):1700. 10.3390/ani10091700. 32962234 10.3390/ani10091700 PMC7552771

[12] Reed LJ, Muench H. A simple method of estimating fifty percent endpoints. Am J Hyg. 1938;27(3):493–7. 10.1093/oxfordjournals.aje.a118408.

[13] Tamer C, Benkaroun J, Kurucay HN, Albayrak H, Weidmann M. Development of a recombinase polymerase amplification assay for viral haemorrhagic septicemia virus. J Fish Dis. 2022;45(8):1065–71. 10.1111/jfd.13629. 35467756 10.1111/jfd.13629

[14] Boxus M, Letellier C, Kerfhofs P. Real Time RT-PCR for the detection and quantitaion of bovine respiratory syncytial virus. J Virol Methods. 2005;125:125–300. 10.1016/j.jviromet.2005.01.008. 15794981 10.1016/j.jviromet.2005.01.008

[15] Vilcek S, Elvander M, Ballagy-Pordany A, Belak S. Development of nested PCR assays for detection of bovine respiratory syncytial virus in clinical samples. J Clin Microbiol. 1994;32(9):2225–31. 10.1128/jcm.32.9.2225-2231.1994. 7814551 10.1128/jcm.32.9.2225-2231.1994 PMC263972

[16] Shacter E, Williams JA, Hinson RM, Sentürker S, Lee YJ. Oxidative stress interferes with cancer chemotherapy: inhibition of lymphoma cell apoptosis and phagocytosis. Blood. 2000;96(1):307–13. 10.1182/blood.V96.1.307 10891466

[17] Baskin H, Ellermann-Eriksen S, Lovmand J, Mogensen SC. Herpes simplex virus type 2 synergizes with interferon-gamma in the induction of nitric oxide production in mouse macrophages through autocrine secretion of tumour necrosis factor-alpha. J Gen Virol. 1997;78(Pt 1):195–203. 10.1099/0022-1317-78-1-195. 9010304 10.1099/0022-1317-78-1-195

[18] Lazniewski M, Dermawan D, Hidayat S, Muchtaridi M, Dawson WK, Plewczynski D. Drug repurposing for identification of potential spike inhibitors for SARS-CoV-2 using molecular docking and molecular dynamics simulations. Methods. 2022;203:498–510. 10.1016/j.ymeth.2022.02.004. 35167916 10.1016/j.ymeth.2022.02.004 PMC8839799

[19] Cohrs RJ, Martin T, Ghahramani P, Bidaut L, Higgins PJ, Shahzad A. Translational Medicine definition by the European Society for Translational Medicine. New Horiz Transl Med. 2015;2:86–8. 10.1016/j.nhtm.2014.12.002.

[20] Garofalo RP, Kolli D, Casola A. Respiratory syncytial virus ınfection: mechanisms of redox control and novel therapeutic opportunities. Antioxid Redox Signal. 2013. 10.1089/ars.2011.4307. 22799599 10.1089/ars.2011.4307 PMC3513983

[21] Aherne W, Bird T, Court SD, Gardner PS, McQuillin J. Pathological changes in virus infections of the lower respiratory tract in children. J Clin Pathol. 1970;23(1):7–18. 10.1136/jcp.23.1.7. 4909103 10.1136/jcp.23.1.7 PMC474401

[22] Haeberle HA, Takizawa R, Casola A, Brasier AR, Dieterich HJ, Van Rooijen N, et al. Respiratory syncytial virus-induced activation of nuclear factor-kappaB in the lung involves alveolar macrophages and toll-like receptor 4-dependent pathways. J Infect Dis. 2002;186(9):1199–206. 10.1086/344644. 12402188 10.1086/344644

[23] Haeberle HA, Casola A, Gatalica Z, Petronella S, Dieterich HJ, Ernst PB, et al. IkappaB kinase is a critical regulator of chemokine expression and lung inflammation in respiratory syncytial virus infection. J Virol. 2004;78(5):2232–41. 10.1128/jvi.78.5.2232-2241.2004. 14963119 10.1128/JVI.78.5.2232-2241.2004 PMC369265

[24] (FDA) U.S. Food and Drug Administration. Montelukast sodium labeling information. Available online: https://www.accessdata.fda.gov/drugsatfda_docs/label/2008/021409s026lbl.pdf. Accessed 20 Jan 2025)

[25] Luedemann M, Stadler D, Cheng CC, Protzer U, Knolle PA, Donakonda S. Montelukast is a dual-purpose inhibitor of SARS-CoV-2 infection and virus-induced IL-6 expression identified by structure-based drug repurposing. Comput Struct Biotechnol J. 2022;20:799–811. 10.1016/j.csbj.2022.01.024. 35126884 10.1016/j.csbj.2022.01.024 PMC8800171

[26] Igde M, Yazici Z. Possible antiviral activity of montelukast against Herpes Simplex Virus type-1 and Human Adeno Virus in vitro. Afr J Microbiol Res. 2012;6(1):197–202. 10.5897/AJMR11.1326.

[27] Chen Y, Wang X, Shi H, Zou P. Montelukast inhibits HCoV-OC43 infection as a viral inactivator. Viruses. 2022;14(5):861. 10.3390/v14050861. 35632604 10.3390/v14050861 PMC9143845

[28] Mulgaonkar N, Wang H, Zhang J, Roundy CM, Tang W, Chaki SP, et al. Montelukast and telmisartan as inhibitors of SARS-CoV-2 omicron variant. Pharmaceutics. 2023;15:1891. 10.3390/pharmaceutics15071891. 37514075 10.3390/pharmaceutics15071891 PMC10385313

[29] Al-Kuraishy HM, Al-Gareeb AI, Almulaiky YQ, Cruz-Martins N, Batiha GE. Role of leukotriene pathway and montelukast in pulmonary and extrapulmonary manifestations of Covid-19: the enigmatic entity. Eur J Pharmacol. 2021;904:174196. 10.1016/j.ejphar.2021.174196. 34004207 10.1016/j.ejphar.2021.174196 PMC8123523

[30] Maeba S, Ichiyama T, Ueno Y, Makata H, Matsubara T, Furukawa S. Effect of montelukast on nuclear factor kappaB activation and proinflammatory molecules. Ann Allergy Asthma Immunol. 2005;94(6):670–4. 10.1016/S1081-1206(10)61326-9. 15984600 10.1016/S1081-1206(10)61326-9

